# Effect of high-dose dexamethasone on perioperative lactate levels and glucose control: a randomized controlled trial

**DOI:** 10.1186/s13054-015-0736-9

**Published:** 2015-02-13

**Authors:** Thomas H Ottens, Maarten WN Nijsten, Jan Hofland, Jan M Dieleman, Miriam Hoekstra, Diederik van Dijk, Joost MAA van der Maaten

**Affiliations:** Department of Anesthesiology and Intensive Care, University Medical Center Utrecht, Mail stop Q.04.2.313, PO Box 85500, Utrecht, 3508 GA The Netherlands; Department of Critical Care, University Medical Center, University of Groningen, Hanzeplein 1, Groningen, RB 9700 The Netherlands; Department of Anesthesiology, Erasmus University Medical Center, ’s Gravendijkwal 230, Rotterdam, CE 3015 The Netherlands; Department of Anesthesiology, University Medical Center Groningen, University of Groningen, Hanzeplein 1, Groningen, RB 9700 The Netherlands

## Abstract

**Introduction:**

Blood lactate levels are increasingly used to monitor patients. Steroids are frequently administered to critically ill patients. However, the effect of steroids on lactate levels has not been adequately investigated. We studied the effect of a single intraoperative high dose of dexamethasone on lactate and glucose levels in patients undergoing cardiac surgery.

**Methods:**

The Dexamethasone for Cardiac Surgery (DECS) trial was a multicenter randomized trial on the effect of dexamethasone 1 mg/kg versus placebo on clinical outcomes after cardiac surgery in adults. Here we report a pre-planned secondary analysis of data from DECS trial participants included at the University Medical Center Groningen. The use of a computer-assisted glucose regulation protocol—Glucose Regulation for Intensive care Patients (GRIP)—was part of routine postoperative care. GRIP aimed at glucose levels of 4 to 8 mmol/L. Primary outcome parameters were area under the lactate and glucose curves over the first 15 hours of ICU stay (AUC_15_). ICU length of stay and mortality were observed as well.

**Results:**

The primary outcome could be determined in 497 patients of the 500 included patients. During the first 15 hours of ICU stay, lactate and glucose levels were significantly higher in the dexamethasone group than in the placebo group: lactate AUC_15_ 25.8 (13.1) versus 19.9 (11.2) mmol/L × hour, *P* <0.001 and glucose AUC_15_ 126.5 (13.0) versus 114.4 (13.9) mmol/L × hour, *P* <0.001. In this period, patients in the dexamethasone group required twice as much insulin compared with patients who had received placebo. Multivariate and cross-correlation analyses suggest that the effect of dexamethasone on lactate levels is related to preceding increased glucose levels. Patients in the placebo group were more likely to stay in the ICU for more than 24 hours (39.2%) compared with patients in the dexamethasone group (25.0%, *P* = 0.001), and 30-day mortality rates were 1.6% and 2.4%, respectively (*P* = 0.759).

**Conclusions:**

Intraoperative high-dose dexamethasone increased postoperative lactate and glucose levels in the first 15 hours of ICU stay. Still, patients in the dexamethasone group had a shorter ICU length of stay and similar mortality compared with controls.

**Trial registration:**

ClinicalTrials.gov NCT00293592. Registered 16 February 2006.

**Electronic supplementary material:**

The online version of this article (doi:10.1186/s13054-015-0736-9) contains supplementary material, which is available to authorized users.

## Introduction

High doses of corticosteroids are often administered to attenuate the inflammatory response to cardiac surgery and cardiopulmonary bypass (CPB) [1]. Administration of a corticosteroid such as methylprednisolone or dexamethasone during cardiac surgery is associated with a reduced duration of postoperative mechanical ventilation and intensive care unit (ICU) and hospital stay, probably because of a lower risk of postoperative pulmonary complications [1-4]. Still, controversy regarding their use remains because of potential important adverse effects. Corticosteroids increase insulin resistance and may increase hyperglycemia and blood glucose variability. The pronounced inflammatory response following cardiac surgery and CPB contributes to decreased insulin secretion and increased insulin resistance. Although too rigid a regulation of blood glucose may increase the risk of hypoglycemia, inadequate glucose control is associated with increased ICU morbidity and mortality [5,6].

Studies on the influence of corticosteroids on lactate levels are scarce, and their results are contradictory [7,8]. Dexamethasone effectively reduces the production of pro-inflammatory cytokines during cardiac surgery and thus may reduce lactate production [7,9] on the one hand, but increases the amount of substrate for lactate formation (pyruvate from glucose) by increasing hepatic glucose release and insulin resistance on the other. Furthermore, dexamethasone increases catecholamine production in the adrenal medulla via its stimulating effect on phenylethanolamine-M-methyltransferase [10]. Catecholamines affect splanchnic perfusion as well as muscle Na-K-ATP-ase activity, Both of which may also lead to increased lactate production [11,12].

Because blood glucose [5,13,14] and lactate levels [15-20] are both well-established and important clinical outcome predictors in ICU patients, including those after cardiac surgery, we investigated the effect of a single intraoperative high dose of dexamethasone on glucose control and lactate levels after cardiac surgery. We studied a subset of patients included in the Dexamethasone for Cardiac Surgery (DECS) trial, a multicenter randomized trial recently carried out in The Netherlands [3]. This trial found a reduced risk of respiratory failure and infections and reduced ICU and hospital length of stay in patients who received dexamethasone. We assessed the effect of dexamethasone on postoperative lactate and glucose levels.

## Methods

### Study design and participants

The DECS trial is a multicenter randomized clinical trial in which 4,494 cardiac surgery patients were randomly assigned to receive either an intraoperative dose of 1 mg/kg dexamethasone or placebo. A detailed description of the design and results of the DECS trial was recently published [3]. The institutional review board (Medical Ethics Committee, in Dutch: Medisch Ethische Toetsingscommissie, METc) of the University Medical Center Utrecht as well as the institutional review board (METc) of the University Medical Center Groningen approved the DECS trial protocol, which adhered to Good Clinical Practice guidelines and relevant national regulations and is registered in the National Institutes of Health Trial Registry (ClinicalTrials.gov identifier: NCT00293592). All participants gave written informed consent.

### Intervention, randomization, and blinding

According to a computer-generated 1:1 randomization scheme, patients received a single intravenous (IV) bolus of dexamethasone (20 mg/mL, 1 mg/kg, maximum 100 mg) or placebo (NaCl 0.9%) immediately after induction of general anesthesia. The trial pharmacy prepared the trial medication in blocks of 40 numbered, indistinguishable vials. Patients, investigators, and all of the patients’ caregivers were blinded to treatment allocation. Surgery, anesthesia, and CPB were conducted without modification for the trial.

### Patient recruitment

The DECS trial recruited adult patients who required elective or urgent cardiac surgery with use of CPB, and excluded off-pump and emergency surgeries and patients with a life expectancy of less than 6 months. For the present study, data of all 500 DECS trial participants who underwent surgery at the University Medical Center Groningen, The Netherlands, were used, because this center routinely uses a validated computer-assisted glucose regulation protocol in the ICU.

### Conduct of anesthesia

Pre-operative cardiac medication was continued until the morning of surgery. Patients were fasted from solid foods for 6 hours and from clear fluids for 2 hours prior to surgery. Patients did not receive supplemental enteral or parental feeding during the pre-operative fasting period. Standard anesthetic management consisted of propofol, sufentanil, and a non-depolarizing muscle relaxant. Etomidate was only incidentally used as an induction agent. Intraoperatively, glucose-free IV maintenance fluids were used in all patients. CPB was performed by using non-pulsatile flow of 2.4 L/min per m^2^, mild hypothermia (32°C to 34°C), and a combined glucose-free colloid/crystalloid priming solution. Myocardial protection consisted of cold blood cardioplegia or cold glucose-free crystalloid solution (Plegisol; Abbott Laboratories, North Chicago, IL, USA). Postoperatively, sedation was continued with propofol and morphine. During the first 24 hours of ICU stay, the standard feeding protocol consisted of 25 g of intravenous glucose (1,000 mL glucose/saline solution) and no additional enteral feeding.

### Lactate and glucose monitoring and insulin therapy

In the ICU, a computer-assisted glucose control protocol named Glucose Regulation for Intensive care Patients (GRIP) was used [21]. GRIP recommends an insulin infusion rate and glucose sampling frequency. The algorithm, which is described in more detail elsewhere [21] and was validated in 2,800 patients [22], is based on both the current glucose level (queried automatically from the hospital information system) and the change in recent glucose values. GRIP-assisted glucose control, targeting for a glucose level between 4 and 8 mmol/L, was applied as part of routine care during the postoperative ICU stay of all patients in this study. According to the protocol, short-acting insulin (NovoRapid, Novo Nordisk, Alphen a/d Rijn, The Netherlands) was administered intravenously via a syringe pump. Intraoperatively, anesthesiologists used a glucose regulation protocol targeting for a blood glucose level below 10 mmol/L but did not use GRIP. Glucose and lactate levels were obtained as part of routine intraoperative blood gas analysis. All blood samples were analyzed on point-of-care ABL Blood Gas Analyzer machines (Radiometer, Copenhagen, Denmark). The analyzers were subject to strict laboratory quality control.

### Outcome measures

The primary outcome was the area under the curve (AUC) of glucose and lactate levels in the first 15 hours of postoperative ICU stay. The AUC was calculated by using the trapezoid rule approximation method, with no threshold for normal values. After 15 hours, most patients were discharged to the surgical ward, where GRIP was not used, glucose levels were measured less frequently, and lactate levels were measured rarely. When patients arrived at the ICU, glucose and lactate levels were always sampled. After that, the GRIP system determined the sampling frequency of glucose and lactate levels, so the sampling frequency differs among patients. This information is presented in Figure S1 (Additional file 1). GRIP suggests a time interval to the next sample, depending on previous glucose values and insulin infusion rate. We therefore interpolated hourly glucose and lactate values to calculate the AUC. Secondary outcomes were total insulin consumption and the hyperglycemic index (HGI) [23]. The HGI is a measure of overall hyperglycemia that is not influenced by hypoglycemic episodes or unequal sampling intervals. It is calculated by dividing the area under the glucose curve that lies above the upper limit of the normal range by the observation time. The HGI was calculated for two cutoff values: 8.0 mmol/L as the upper end of our target glucose range and 6.0 mmol/L according to the original method of calculation described by Vogelzang *et al*. [23]. Intraoperatively, glucose and lactate were routinely sampled at least three times: before, during, and after CPB. Finally, we report ICU length of stay, ICU readmissions, inotropic or vasopressor support requirement during the ICU stay, postoperative surgical site infections, gastrointestinal hemorrhages, length of hospital stay, and 30-day mortality.

### Regression model

Because the effect of dexamethasone on lactate levels is not well understood, we evaluated the role of glucose as an intermediate in the relation between dexamethasone and postoperative lactate levels. In a multivariate linear regression model, we assessed the effect of dexamethasone on postoperative lactate levels (AUC of the first 15 hours of postoperative ICU stay) and added postoperative glucose levels (AUC of the first 15 hours of postoperative ICU stay) to the model as a covariable.

### Subgroup analyses

Two subgroup analyses were performed. We calculated the main outcome measures separately for patients with diabetes. The findings of this analysis are presented in Table 1. A *post hoc* regression analysis on the effect of beta-blocker use on the primary outcome was carried out because of a significant baseline difference between the groups.

**Table 1:** **Postoperative glucose control, insulin requirement, and lactate level in a subgroup of patients with diabetes**

**Outcome measure** ^**a**^	**Dexamethasone ** **(n = 62)**	**Placebo ** **(n = 58)**	***P*** **value**
Glucose AUC_15_ in mmol/L × hour	132.8 (16.2)	124.8 (16.5)	0.009
Glucose level in mmol/L at postoperative ICU admission	10.0 (2.5)	8.0 (2.3)	<0.001
Hyperglycemic index in mmol/L^b^ (cutoff >6.0 mmol/L)	2.70 (1.00)	2.21 (1.00)	0.009
Hyperglycemic index in mmol/L^c^ (cutoff >8.0 mmol/L)	1.14 (0.91)	0.77 (0.76)	0.017
Hypoglycemia incidence, N (%)^d^	0 (0)	3 (5.2)	0.110
Lactate AUC_15_ in mmol/L × hour	25.9 (9.7)	22.1 (16.2)	0.119
Lactate level in mmol/L at postoperative ICU admission	2.1 (1.0)	1.7 (0.7)	0.007
Insulin consumption in IU at first 15 hours of ICU admission	46.6 (22.9)	28.3 (19.7)	<0.001

To convert glucose to mg/dL, multiply by 18.02. To convert lactate to mg/dL, multiply by 9.01. ^a^All data refer to the first 15 postoperative hours and are presented as mean (standard deviation) unless otherwise indicated. ^b^Calculated by dividing the part of the glucose AUC_15_ (area under the curve in the first 15 hours of postoperative intensive care unit stay) that lies above 6.0 mmol/L by observation time. ^c^Calculated by dividing the part of the glucose AUC_15_ that lies above 8.0 mmol/L by observation time. ^d^Defined as any glucose level of less than 4.0 mmol/L in the first 15 postoperative hours. ICU, intensive care unit.

### Statistical analysis

The primary outcome was the AUC_15_ (first 15 postoperative hours) of postoperative glucose and lactate levels. To assess normality of distribution, frequency distribution histograms with normality curves, as well as Levene’s test, were used. We compared the AUC_15_ by using the Student’s *t* test. If patients were discharged from the ICU before the 15-hour observation period, the last glucose and lactate value was carried forward to calculate the AUC. To compare ICU length of stay between groups, the log-rank test was used. To compare other continuous outcomes, we used the Student’s *t* test or the Mann-Whitney *U* test when appropriate. For dichotomous outcomes, we calculated absolute risk difference and relative risk with 95% confidence interval and compared proportions by using the χ^2^ test. We considered a two-tailed *P* value of less than 0.05 to be statistically significant for the primary outcome. For the comparison of the other outcome measures, we considered a two-tailed *P* value of less than 0.001 to be statistically significant. IBM SPSS 22 (IBM Corporation, Armonk, NY, USA) was used for all analyses.

## Results

### Study population

**Figure 1:**
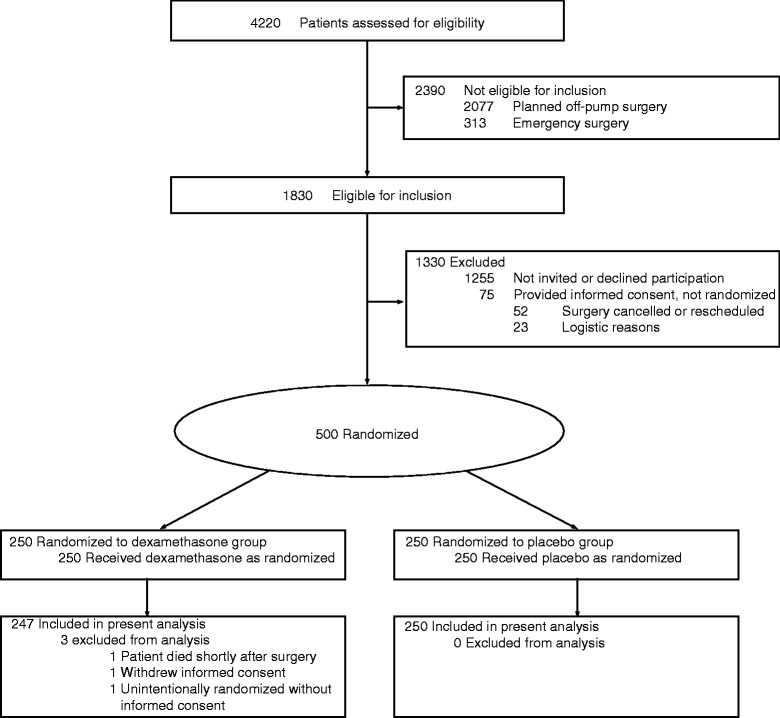
**Enrollment flowchart.** The Dexamethasone for Cardiac Surgery (DECS) trial was a multicenter study with 4,494 participants who were treated at eight participating hospitals in The Netherlands. This DECS trial substudy concerned patients who underwent cardiac surgery at the University Medical Center Groningen.

Between February 2008 and July 2011, 500 patients were enrolled and randomly assigned in the DECS trial at the University Medical Center Groningen. One patient withdrew informed consent during the first year of follow-up. One patient was unintentionally randomly assigned without having provided informed consent. Both patients were excluded from the analysis. One patient died within 2 hours after ICU admission and was excluded from the primary outcome analysis. Postoperatively, seven patients were not admitted to the cardiothoracic ICU but to other ICUs where lactate levels were not routinely measured. For these patients, only the AUC_15_ for glucose could be calculated. The enrollment flowchart of this study is shown in Figure 1. Patients’ baseline demographic, clinical, and surgical characteristics were similar between the study groups, except for pre-operative beta-blocker use (dexamethasone group 69% versus placebo group 60%, *P* = 0.046) (Table 2).

**Table 2:** **Demographic, clinical, and surgical characteristics of the dexamethasone and placebo groups** ^**a**^

**Characteristics**	**Dexamethasone ** **(n = 247)**	**Placebo ** **(n = 250)**	***P*** **value**
Demographics			
Male sex	158 (64)	159 (64)	0.83
Age in years, mean (SD)	66.8 (11.7)	66.5 (12.0)	0.78
Weight in kg, mean (SD)	82.1 (14.0)	82.3 (14.3)	0.91
Height in cm, mean (SD)	173 (9.3)	173 (9.2)	0.85
Comorbidities			
Hypertension	146 (59)	151 (60)	0.73
Diabetes			
Insulin-dependent	12 (5)	18 (7)	0.27
Non-insulin-dependent	50 (20)	40 (16)	0.23
Treatment for pulmonary disease	48 (19)	57 (23)	0.35
Previous cerebrovascular event			
Stroke	7 (3)	12 (5)	0.55
Transient ischemic attack	12 (5)	14 (6)	0.70
Peripheral vascular disease	37 (15)	34 (14)	0.67
Pre-operative serum creatinine in mg/dL, mean (SD)	1.02 (0.44)	1.02 (0.42)	0.89
Cardiac status			
Recent myocardial infarction (<90 days)	39 (16)	26 (10)	0.08
Left ventricular function^b^			
Moderate	82 (33)	93 (37)	0.33
Poor	15 (6)	14 (6)	0.83
EuroScore, median (IQR)^c^	6 (4-8)	6(4-8)	0.37
Pre-operative medication			
Beta-blocker	171 (69)	151 (60)	0.05
Statin	142 (57)	125 (50)	0.13
Corticosteroid	21 (9)	17 (7)	0.83
Type of surgery			0.25
Isolated CABG	74 (30)	57 (23)	0.08
CABG plus valve	65 (26)	66 (26)	0.96
Single valve	95 (38)	104 (42)	0.45
Multiple valves	8 (3)	16 (6)	0.10
Other procedures	6 (2)	7 (3)	0.79
Repeat surgery	17 (7)	19 (8)	0.75
Duration of the procedure in minutes, mean (SD)	262 (91)	265 (86)	0.51
Duration of extracorporeal circulation in minutes, mean (SD)	147 (74)	149 (69)	0.70
Duration of aortic cross-clamping in minutes, mean (SD)	95 (49)	99 (46)	0.43
Type of anesthesia maintenance			0.92
Propofol	133 (53.6)	132 (52.8)	
Volatile	115 (46.4)	118 (47.2)	
Use of cell-saving device	17 (7)	23 (9)	0.34
Use of antifibrinolytic agent	179 (72)	179 (72)	0.72

International System conversion: To convert creatinine to μmol/L, multiply by 88.4. ^a^Data presented as number (percentage) unless otherwise indicated. ^b^Definition of left ventricular function classes: moderate, ejection fraction of 30% to 50%; and poor, ejection fraction of less than 30% [24]. ^c^Higher EuroScores indicate increased risk of perioperative mortality [24]. CABG, coronary artery bypass grafting; IQR, interquartile range; SD, standard deviation.

### Outcomes

**Figure 2:**
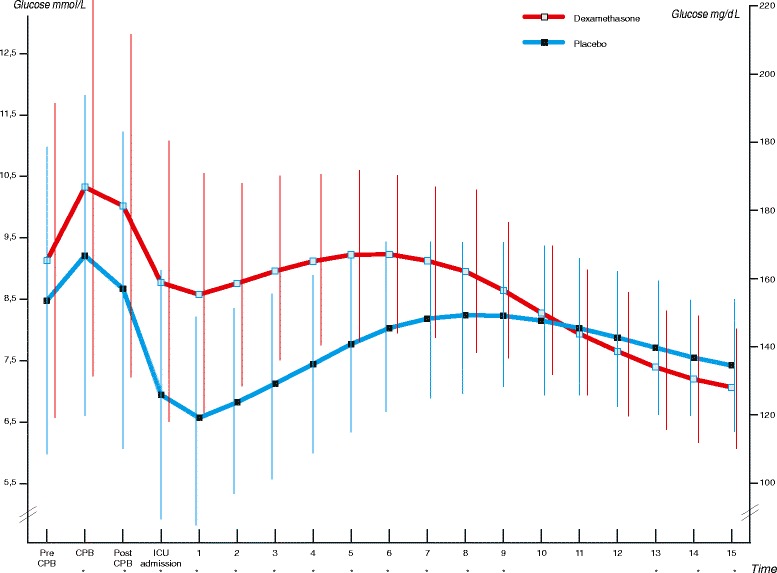
**Intraoperative and postoperative glucose levels.** Mean (standard deviation) glucose levels are shown. The mean AUC_15_ (area under the curve in the first 15 hours of postoperative intensive care unit stay) values were 127 mmol/L × hour for the dexamethasone group and 114 mmol/L × hour for the placebo group (*P* <0.001). To convert glucose to mg/dL, multiply by 18.02.

**Figure 3:**
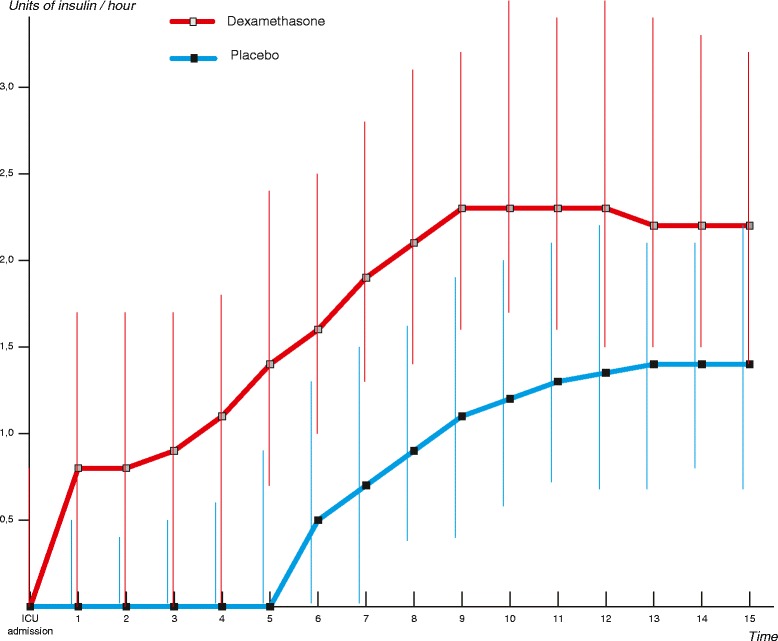
**Postoperative insulin infusion rate, as prescribed by the Glucose Regulation for Intensive care Patients (GRIP) system.** Median (interquartile range) infusion rate in units of short-acting insulin/hour is shown. Mean cumulative insulin doses (first 15 hours of intensive care unit stay) were 32.0 units in the dexamethasone group and 16.3 units in the placebo group (*P* <0.001).

**Figure 4:**
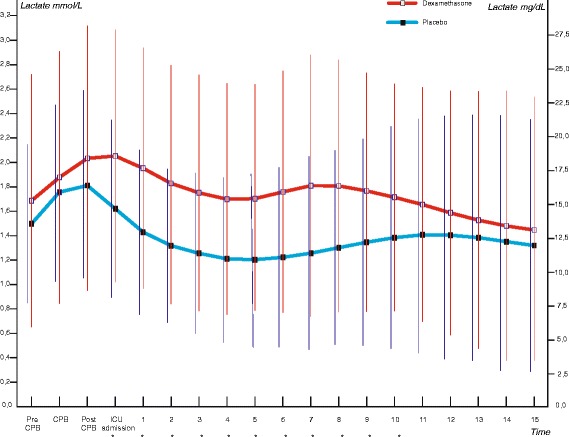
**Intraoperative and postoperative lactate levels.** Mean (standard deviation) lactate levels are shown. Mean lactate AUC_15_ values were 26 mmol/L × hour in the dexamethasone group and 20 mmol/L × hour in the placebo group (*P* <0.001). To convert lactate to mg/dL, multiply by 9.01. *Difference between groups statistically significant (*P* <0.001 after Bonferroni correction). AUC_15_, area under the curve in the first 15 hours of postoperative intensive care unit stay; CPB, cardiopulmonary bypass; ICU, intensive care unit.

**Figure 5:**
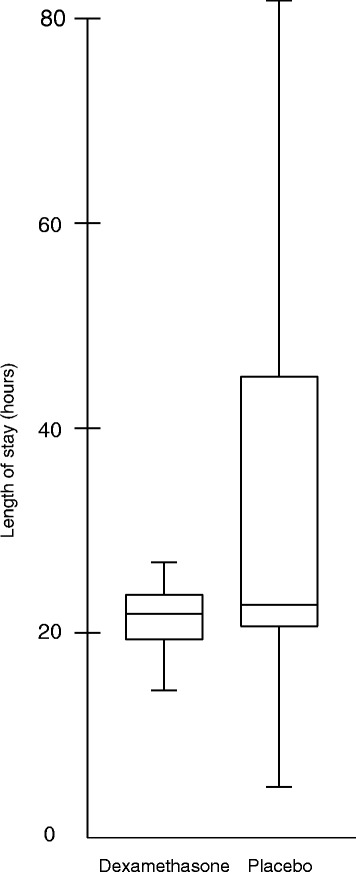
**Length of postoperative intensive care unit stay in the dexamethasone and placebo groups.** Box-plot of postoperative intensive care unit length of stay is shown. Whiskers represent the last datum within 1.5 times the interquartile range. Median (interquartile range) lengths of stay were 21.9 (4.3) hours in the dexamethasone group and 22.7 (24.5) hours in the placebo group (*P* <0.001) (log-rank test). Note the difference between the median and mean lengths of stay (Table 3).

Table 3 shows the primary outcome of the study. The mean areas under the glucose curve during the first 15 hours of postoperative ICU admission were 126.5 mmol/L × hour in the dexamethasone group and 114.4 mmol/L × hour in the placebo group (*P* <0.001) (Figure 2; individual curves in Figure S2, Additional file 1). In the first 15 hours of postoperative ICU admission, patients who received dexamethasone required approximately twice as much insulin compared with patients who received placebo (32.0 versus 16.3 units, *P* <0.001) (Figure 3). More frequent insulin infusion rate adjustments were necessary to control glucose levels in patients who received dexamethasone (Figure S1 and Figure S2, Additional file 1). The mean areas under the lactate curve during the first 15 hours after postoperative ICU admission were 25.8 mmol/L × hour in the dexamethasone group and 19.9 mmol/L × hour in the placebo group (*P* <0.001) (Figure 4; individual curves in Figure S2, Additional file 1). Patients in the dexamethasone group had a significantly shorter ICU length of stay: dexamethasone median (interquartile range, or IQR) 21.9 (4.3) versus placebo 22.7 (24.5) hours, *P* <0.001 (Figure 5). Although the difference in median length of stay is less than 1 hour, more patients in the placebo group were not ready for discharge from the ICU at 24 hours postoperatively. The numbers of patients who stayed in the ICU for 24 hours or longer were 62 (25.0%) in the dexamethasone group and 98 (39.2%) in the placebo group (*P* = 0.001). At 30-day follow-up, four patients in the dexamethasone group (1.6%) and six patients in the placebo group (2.4%) had died.

**Table 3:** **Postoperative outcomes in the dexamethasone and placebo groups**

**Outcome measure** ^**a**^	**Dexamethasone**	**Placebo**	***P*** **value**
Glucose AUC_15_ in mmol/L × hour	126.5 (13.0)	114.4 (13.9)	<0.001
Glucose level in mmol/L at postoperative ICU admission	8.7 (2.3)	6.9 (2.0)	<0.001
Hyperglycemic index in mmol/L^b^ (cutoff >6.0 mmol/L)	1.97 (0.85)	1.28 (0.81)	<0.001
Hyperglycemic index in mmol/L^c^ (cutoff >8.0 mmol/L)	0.74 (0.69)	0.35 (0.51)	<0.001
Hypoglycemia incidence, N (%)^d^	3 (1.2)	20 (8.0)	<0.001
Lactate AUC_15_ in mmol/L × hour	25.8 (13.1)	19.9 (11.2)	<0.001
Lactate level in mmol/L at postoperative ICU admission	2.1 (1.0)	1.6 (0.7)	<0.001
Insulin consumption in IU at first 15 hours of ICU admission	32.0 (19.9)	16.3 (14.5)	<0.001
ICU length of stay in hours, median (IQR)	21.9 (19.3-23.7)	22.7 (20.6-45.0)	0.001
ICU length of stay ≥24 hours, N (%)	62 (25.0)	98 (39.2)	0.001
ICU readmissions, N (%)	13 (5.2)	10 (4.0)	0.51
Hospital length of stay in days, median (IQR)	10 (8-16)	11 (8-16)	0.53
Postoperative inotropic or vasopressor requirement, N (%)	125 (50.6)	144 (57.6)	0.14
Postoperative surgical site infections, N (%)	5 (2.0)	6 (2.4)	0.77
Postoperative gastrointestinal hemorrhage, N (%)	3 (1.2)	1 (0.4)	0.37
30-day mortality, N (%)^e^	4 (1.6)	6 (2.4)	0.76

To convert glucose to mg/dL, multiply by 18.02. To convert lactate to mg/dL, multiply by 9.01. ^a^All data refer to the first 15 postoperative hours and are presented as mean (standard deviation) unless otherwise indicated. ^b^Calculated by dividing the part of the glucose AUC_15_ (area under the curve in the first 15 hours of postoperative intensive care unit stay) that lies above 6.0 mmol/L by observation time. ^c^Calculated by dividing the part of the glucose AUC_15_ that lies above 8.0 mmol/L by observation time. ^d^Defined as any glucose level of less than 4.0 mmol/L in the first 15 postoperative hours. ^e^Defined as any use of adrenalin, noradrenalin, dopamine, dobutamine, or milrinone during the first 24 hours of intensive care unit stay. ICU, intensive care unit; IQR, interquartile range.

Regression analysis shows that the association between treatment with dexamethasone and increase postoperative lactate levels is no longer statistically significant when postoperative glucose level is added as a covariable. This suggests that dexamethasone exerts its effect on postoperative lactate levels mainly via glucose. Regression betas and *P* values are presented in Table S1 in Additional file 1.

*Post hoc* bivariate cross-correlation analysis shows that glucose levels had the strongest correlation with lactate levels 2 hours later. These data are presented in Table S2 in Additional file 1.

In the subgroup of patients with diabetes (n = 120), a similar effect of dexamethasone on glucose AUC_15_ was observed, compared with patients without diabetes. However, in patients with diabetes, the lactate AUC_15_ did not differ (Table 1). Pre-operative beta-blocker use was independently associated with a small increase in postoperative glucose levels (beta 3.720, *P* = 0.003) but not lactate levels (beta 1.921, *P* = 0.096).

## Discussion

This randomized trial of 497 cardiac surgery patients showed that a single intraoperative high dose of dexamethasone leads to significantly increased lactate and glucose levels in the early postoperative period, with the use of a computer-assisted glucose control system. In the early postoperative period, the average insulin requirement was two times the average amount required in patients who received placebo. Still, patients who received dexamethasone were more likely to be discharged from the ICU within 24 hours after surgery compared with patients who received placebo, as previously observed in the parent trial [3]. Bivariate correlation analysis and multivariate regression analysis suggest that the higher glucose levels in patients who received dexamethasone may largely be responsible for the higher lactate levels.

Lactate measurement and corticosteroid administration are widely used, and both arguably have a unique position in critical care. Lactate has emerged as one of the most useful prognostic laboratory parameters to predict poor outcome [15,18,19]. Recent studies have demonstrated that lactate can be used to monitor the adequacy of circulatory management and that treatment targeted towards reducing lactate levels has clinical benefit in ICU patients [25]. On the therapeutic side, corticosteroid therapy is probably the most debated intervention in critical care [1,2,4,26]. Against this background, it is surprising that relatively little evidence exists on the direct impact of corticosteroids upon lactate levels. Other than the aforementioned small studies from 1997 and 2003, we are not aware of other randomized double-blind placebo-controlled studies that have studied the effect of high-dose corticosteroids on postoperative lactate levels [7,8].

Our observation has practical consequences regarding the prognostic value of moderately increased lactate and glucose levels. In this study, patients who received dexamethasone had higher lactate levels, required more insulin, and had higher glucose levels, but the parent trial (n = 4,494) showed that dexamethasone reduced ICU length of stay and that there was no effect of dexamethasone on major adverse events (7.0% after dexamethasone versus 8.5% after placebo, *P* = 0.07) [3]. The present substudy was underpowered to evaluate the effect of dexamethasone on major adverse events but confirmed the shorter length of stay in the ICU found in the dexamethasone group in the parent trial.

**Figure 6:**
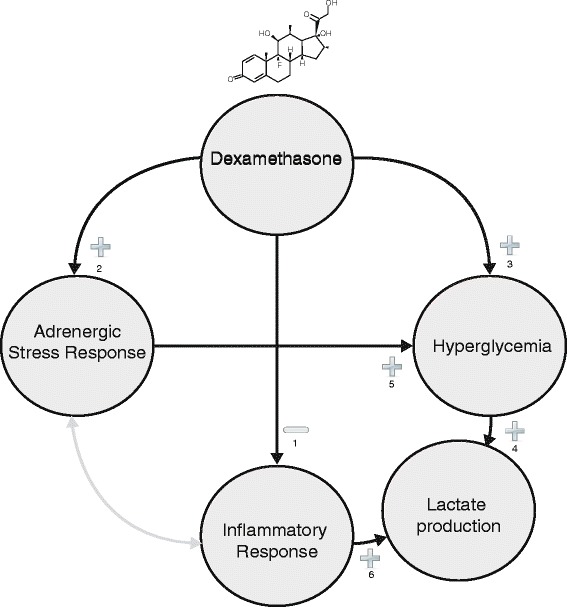
**The influence of dexamethasone on glucose and lactate levels. (1)** Dexamethasone reduces production of pro-inflammatory cytokines. **(2)** In the adrenergic medulla, dexamethasone increases catecholamine production. **(3)** Dexamethasone stimulates hepatic glucose production and increases insulin resistance. **(4)** Glucose can be metabolized to pyruvate and to lactate via lactate dehydrogenase. **(5)** Catecholamine stimulation can increase lactate production via increased Na-K-pump activity [10-12,27-31].

The pleiotropic effects of dexamethasone and the pleiotropic causes of hyperlactatemia make it impossible to determine the dominant pathways involved. Figure 6 shows several potential stimulatory and inhibitory pathways that may have played a role in the induction of higher lactate levels in patients who received dexamethasone and the strong relation of this effect with high glucose levels. Dexamethasone may reduce lactate production by inhibiting the synthesis of pro-inflammatory cytokines associated with increased lactate production [7,9,12,27-29]. This effect may have been outweighed by dexamethasone’s effects on the adrenergic stress response, which increases blood lactate directly through increased catecholamine production in the adrenal medulla. Indirectly, dexamethasone increases lactate production through its effect on insulin resistance and hepatic production of glucose, which increases blood glucose, which is a substrate for lactate production [10-12]. In this study, multivariate regression suggests that the effect of dexamethasone on lactate production is dependent on its hyperglycemic effect.

Whether the positive relation between lactate and corticosteroids that we observed is restricted to cardiac surgery patients who receive high-dose dexamethasone or whether it is also present in, for example, sepsis patients who receive comparatively lower doses of hydrocortisone is not known. Detailed analysis of glucose and lactate measurements in sepsis patients might address this question. In a recent retrospective study on the relation of hyperlactatemia and hyperglycemia with outcome in a cohort of nearly 8,000 ICU patients, it was observed that hyperlactatemia and hyperglycemia each had a strong relation with mortality but that the independent relation of hyperglycemia with outcome virtually disappeared when hyperlactatemia and hyperglycemia were included together. The investigators suggest that this observation indicates that stress is a common cause for increases of both lactate and glucose [32]. In the case of dexamethasone administration, measurements of adrenergic stress hormone and cytokine levels might further elucidate the pathways by which dexamethasone or other corticosteroids influence glucose and lactate levels. Bivariate cross-correlation analysis (presented in Table S2, Additional file 1) supports the suggestion of a causal relation as reflected by the time course of the mean glucose and lactate curves, namely that the glucose peak induces a lactate peak a few hours later. Future studies with continuous monitoring of circulating glucose and lactate as well as a sampling of mediators such as epinephrine and registration of exogenous catecholamines may further clarify the apparent temporal and potential causal relation between the (postoperative) glucose peak and subsequent lactate peak.

Several limitations apply to our study. This study has a relatively short follow-up time. Most patients leave the ICU within 24 hours, limiting the time frame for highly frequent glucose and lactate sampling, although as the temporal data show, the period of maximal insulin resistance has clearly passed by then.

In this study population, the 30-day mortality was 2%, somewhat higher than the overall 30-day mortality in the DECS trial (1.5%). This can be explained by a difference in case mix, reflected by the higher median Euroscore of the patients operated at the University Medical Center Groningen compared with the overall Euroscore in the DECS trial: 6 (IQR 4 to 8) versus 5 (IQR 3 to 7).

This study was performed at an ICU with a validated computer-assisted glucose regulation protocol. We expect that, on average, glucose levels in both groups would have been higher if less intensive glucose control had been applied. The GRIP system advised much larger insulin doses to patients who received dexamethasone. We therefore expect that a larger difference between the glucose levels of patients who received dexamethasone versus those who received placebo would have occurred in an environment with less intensive glucose control. Because of the double blinding, modified glucose regulation algorithms for patients receiving steroids could not be used. Our results show that specific glucose regulation algorithms for cardiac surgery patients receiving corticosteroids are important to prevent early postoperative hyperglycemia.

The higher glucose levels in patients in the dexamethasone group may have indicated the treatment allocation in some patients. Still, there was a considerable overlap of postoperative glucose levels in the different treatment groups (Figure S2, Additional file 1), and insulin dose changes were prescribed by the GRIP computer system and applied by nurses, who were not involved in the analysis of the data.

A final important limitation of our study is the difference in the number of patients who used beta-blockers at baseline, despite adequate randomization. Pre-operative beta-blocker use was associated with a small but statistically significant increase in postoperative glucose levels but not lactate levels. More patients randomly assigned toward receiving dexamethasone used beta-blockers pre-operatively. This may have contributed to a part of the observed effect of dexamethasone on postoperative glucose levels.

## Conclusions

In this randomized clinical trial of 498 patients, the administration of intraoperative high-dose dexamethasone was associated with significantly higher postoperative lactate and glucose levels after cardiac surgery but was associated with decreased ICU length of stay.

## Key messages

- Dexamethasone increases postoperative glucose levels and insulin requirements in patients who undergo cardiac surgery.
- Dexamethasone increases postoperative lactate levels in patients who undergo cardiac surgery.
- Our data suggest that the increase in lactate levels may be directly related to the increased glucose levels.
- Although high-dose dexamethasone therapy caused an increase in glucose and lactate levels, the treatment was not associated with increased mortality and was associated with shorter intensive care unit length of stay.
- If dexamethasone is administered to cardiac surgery patients, clinicians should anticipate higher insulin requirements and be aware that small increases in lactate levels in such patients are not necessarily related to poor outcome.

## Additional file

Additional file 1: Figure S1. Lactate and glucose sampling frequency and number of insulin infusion rate adjustments in the first 15 hours of postoperative intensive care unit stay. The figure shows a graphical representation of difference in sampling frequency of lactate and glucose and insulin infusion rate adjustments between the dexamethasone and placebo groups during the observation period of the study. **Figure S2.** Individual measurements of glucose, lactate, and insulin infusion rate in the dexamethasone and placebo groups. The figure shows a graphical representation of unadjusted (individual) measurements of glucose and lactate levels and the actual insulin infusion rate during the observation period of the study. **Table S1.** Multivariate linear regression model of the effect of dexamethasone on postoperative lactate levels. The table shows the linear regression models of the effect of treatment allocation (to dexamethasone or placebo) and postoperative glucose levels (area under the curve in the first 15 hours of postoperative intensive care unit stay (AUC_15_) on postoperative lactate levels (AUC_15_). **Table S2.** Bivariate correlation matrix of glucose and lactate levels in the first 15 hours of postoperative intensive care unit (ICU) stay. The table shows the bivariate correlation coefficients of glucose and lactate levels at each time point in the first 15 hours of postoperative ICU stay.

## Abbreviations

AUC: area under the curve
AUC_15_: area under the curve in the first 15 hours of postoperative intensive care unit stay
CPB: cardiopulmonary bypass
DECS: Dexamethasone for Cardiac Surgery
GRIP: Glucose Regulation for Intensive care Patients
HGI: hyperglycemic index
ICU: intensive care unit
IQR: interquartile range
IV: intravenous
METc: Medisch Ethische Toetsingscommissie (Medical Ethics Committee)

## References

[1] Dieleman JM, van Paassen J, Van Dijk D, Arbous MS, Kalkman CJ, Vandenbroucke JP (2011). Prophylactic corticosteroids for cardiopulmonary bypass in adults. Cochrane Database Syst Rev..

[2] Whitlock RP, Chan S, Devereaux PJ, Sun J, Rubens FD, Thorlund K (2008). Clinical benefit of steroid use in patients undergoing cardiopulmonary bypass: a meta-analysis of randomized trials. Eur Heart J..

[3] Dieleman JM, Nierich AP, Rosseel PM, van der Maaten JM, Hofland J, Diephuis JC (2012). Intraoperative high-dose dexamethasone for cardiac surgery: a randomized controlled trial. JAMA..

[4] Cappabianca G, Rotunno C, de Luca Tupputi Schinosa L, Ranieri VM, Paparella D (2011). Protective Effects of Steroids in Cardiac Surgery: A Meta-Analysis of Randomized Double-Blind Trials. J Cardiothorac Vasc Anesth.

[5] Van den Berghe G, Wouters P, Weekers F, Verwaest C, Bruyninckx F, Schetz M (2001). Intensive insulin therapy in the critically ill patients. N Engl J Med..

[6] Finfer S, Chittock DR, Su SY, Blair D, Foster D, NICE-SUGAR Study Investigators (2009). Intensive versus conventional glucose control in critically ill patients. N Engl J Med.

[7] Kilger E, Weis F, Briegel J, Frey L, Goetz AE, Reuter D (2003). Stress doses of hydrocortisone reduce severe systemic inflammatory response syndrome and improve early outcome in a risk group of patients after cardiac surgery. Crit Care Med..

[8] Mayumi H, Zhang QW, Nakashima A, Masuda M, Kohno H, Kawachi Y (1997). Synergistic immunosuppression caused by high-dose methylprednisolone and cardiopulmonary bypass. Ann Thorac Surg..

[9] Taylor DJ, Faragher EB, Evanson JM (1992). Inflammatory cytokines stimulate glucose uptake and glycolysis but reduce glucose oxidation in human dermal fibroblasts in vitro. Circ Shock..

[10] Wurtman RJ, Axelrod J (1966). Control of enzymatic synthesis of adrenaline in the adrenal medulla by adrenal cortical steroids. J Biol Chem.

[11] Luchette FA, Friend LA, Brown CC, Upputuri RK, James JH (1998). Increased skeletal muscle Na+, K + -ATPase activity as a cause of increased lactate production after hemorrhagic shock. J Trauma..

[12] Landow L (1993). Splanchnic lactate production in cardiac surgery patients. Crit Care Med..

[13] Maerz LL, Akhtar S (2011). Perioperative glycemic management in 2011: paradigm shifts. Curr Opin Crit Care..

[14] Akhtar S, Barash PG, Inzucchi SE (2010). Scientific principles and clinical implications of perioperative glucose regulation and control. Anesth Analg..

[15] Bakker J, Nijsten M, Jansen TC (2013). Clinical use of lactate monitoring in critically ill patients. Ann Intensive Care..

[16] Maillet J-M, Le Besnerais P, Cantoni M, Nataf P, Ruffenach A, Lessana A (2003). Frequency, risk factors, and outcome of hyperlactatemia after cardiac surgery. Chest..

[17] Abramson D, Scalea TM, Hitchcock R, Trooskin SZ, Henry SM, Greenspan J (1993). Lactate clearance and survival following injury. J Trauma..

[18] Bakker J, Gris P, Coffernils M, Kahn RJ, Vincent JL (1996). Serial blood lactate levels can predict the development of multiple organ failure following septic shock. Am J Surg..

[19] Jansen TC, Van Bommel J, Woodward R, Mulder PG, Bakker J (2009). Association between blood lactate levels, Sequential Organ Failure Assessment subscores, and 28-day mortality during early and late intensive care unit stay: a retrospective observational study. Crit Care Med..

[20] McNelis J, Marini C, Jurkiewicz A, Szomstein S, Simms H, Ritter G (2001). Prolonged lactate clearance is associated with increased mortality in the surgical intensive care unit. Am J Surg..

[21] Vogelzang M, Zijlstra F, Nijsten MW (2005). Design and implementation of GRIP: a computerized glucose control system at a surgical intensive care unit. BMC Med Inform Decis Mak..

[22] Vogelzang M, Loef BG, Regtien JG, Van der Horst IC, Van Assen H, Zijlstra F (2008). Computer-assisted glucose control in critically ill patients. Intensive Care Med..

[23] Vogelzang M, Van der Horst IC, Nijsten MW (2004). Hyperglycaemic index as a tool to assess glucose control: a retrospective study. Crit Care..

[24] Nashef SA, Roques F, Michel P, Gauducheau E, Lemeshow S, Salamon R (1999). European system for cardiac operative risk evaluation (EuroSCORE). Eur J Cardiothorac Surg..

[25] Jansen TC, Van Bommel J, Schoonderbeek FJ, Visser SJ, van der Klooster JM, Lima AP (2010). Early Lactate-Guided Therapy in Intensive Care Unit Patients. Am J Respir Crit Care Med..

[26] Annane D, Bellissant E, Bollaert P-E, Briegel J, Confalonieri M, De Gaudio R (2009). Corticosteroids in the treatment of severe sepsis and septic shock in adults: a systematic review. JAMA..

[27] El-Azab SR (2002). Dexamethasone decreases the pro- to anti-inflammatory cytokine ratio during cardiac surgery. Br J Anaesthesia..

[28] Jansen NJ, van Oeveren W, van den Broek L, Oudemans-Van Straate HM, Stoutenbeek CP, Joen MC (1991). Inhibition by dexamethasone of the reperfusion phenomena in cardiopulmonary bypass. J Thorac Cardiovasc Surg..

[29] Karaman K, Bostanci EB, Aksoy E, Ulas M, Yigit T, Erdemli MO (2013). Effects of dexamethasone and pheniramine hydrogen maleate on stress response in patients undergoing elective laparoscopic cholecystectomy. Am J Surg..

[30] Kilger E, Weis FC, Goetz AE, Frey L, Kesel K, Schütz A (2001). Intensive care after minimally invasive and conventional coronary surgery: a prospective comparison. Intensive Care Med..

[31] Harris DN, Bailey SM, Smith PL, Taylor KM, Oatridge A, Bydder GM (1993). Brain swelling in first hour after coronary artery bypass surgery. Lancet..

[32] Kaukonen K-M, Bailey M, Egi M, Orford N, Glassford NJ, Marik PE (2014). Stress hyperlactatemia modifies the relationship between stress hyperglycemia and outcome: a retrospective observational study. Crit Care Med..

