# Supplementary material for: Effect of high-dose dexamethasone on perioperative lactate levels and glucose control: a randomized controlled trial
Source: Crit Care. 2015 Feb 13;19(1):41. doi: 10.1186/s13054-015-0736-9 (PMC4350954; doi:10.1186/s13054-015-0736-9)
Supplement: Additional file 1: Figure S1. — Lactate and glucose sampling frequency and number of insulin infusion rate adjustments in the first 15 hours of postoperative intensive care unit stay. The figure shows a graphical representation of difference in sampling frequency of lactate and glucose and insulin infusion rate adjustments between the dexamethasone and placebo groups during the observation period of the study. Figure S2. Individual measurements of glucose, lactate, and insulin infusion rate in the dexamethasone and placebo groups. The figure shows a graphical representation of unadjusted (individual) measurements of glucose and lactate levels and the actual insulin infusion rate during the observation period of the study. Table S1. Multivariate linear regression model of the effect of dexamethasone on postoperative lactate levels. The table shows the linear regression models of the effect of treatment allocation (to dexamethasone or placebo) and postoperative glucose levels (area under the curve in the first 15 hours of postoperative intensive care unit stay (AUC15) on postoperative lactate levels (AUC15). Table S2. Bivariate correlation matrix of glucose and lactate levels in the first 15 hours of postoperative intensive care unit (ICU) stay. The table shows the bivariate correlation coefficients of glucose and lactate levels at each time point in the first 15 hours of postoperative ICU stay. [file 13054_2015_736_MOESM1_ESM.pdf]

Effect of high-dose dexamethasone on perioperative lactate levels and glucose control: a randomized controlled trial.

Additional Figures and Tables

Additional Figure 1: Lactate (a) and glucose (b) sampling frequency and number of insulin infusion rate readjustments (c) in the first 15 hours of postoperative ICU stay.

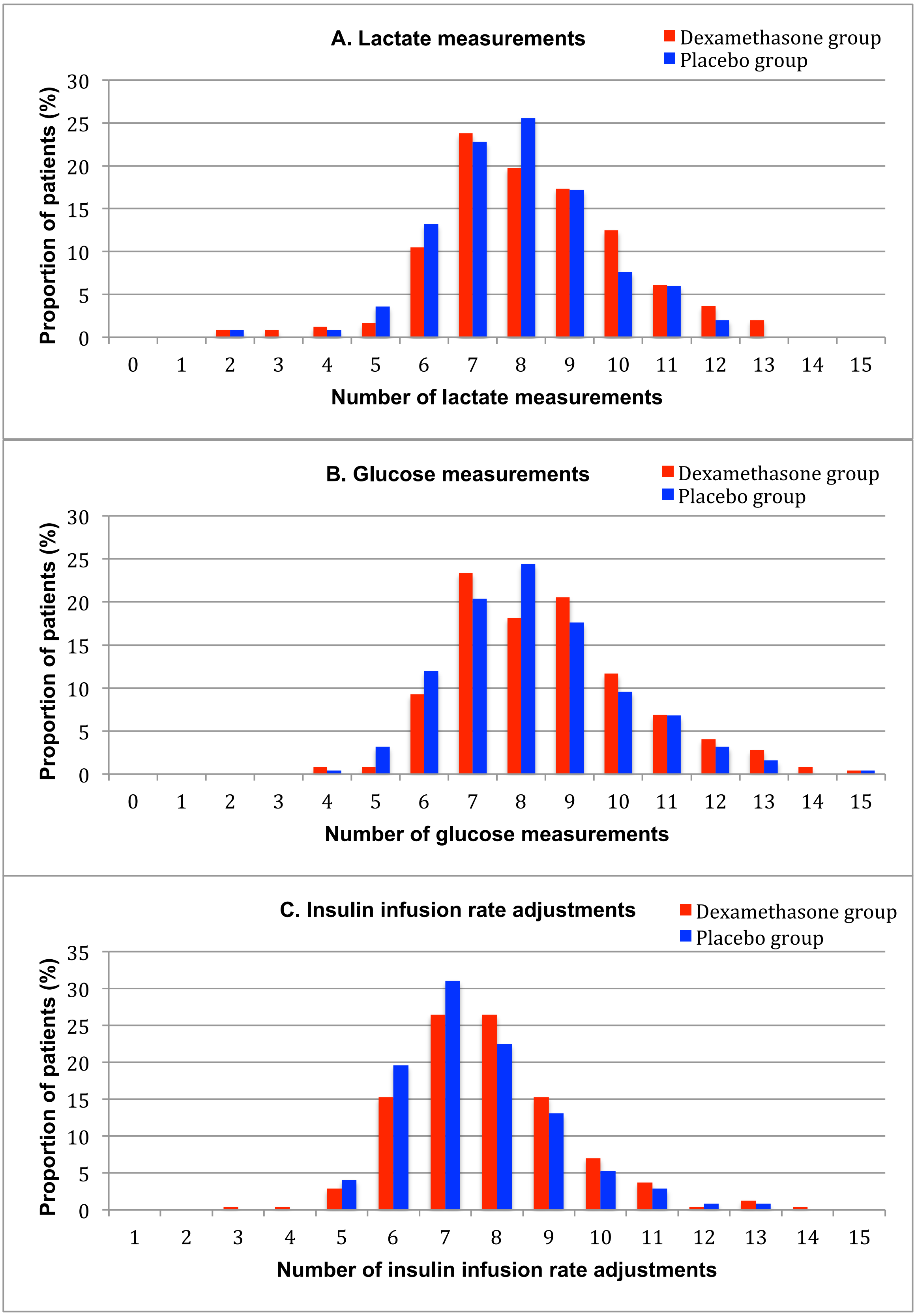

Additional Figure 2: Individual measurements of glucose, lactate and insulin infusion rate in the dexamethasone and placebo groups.

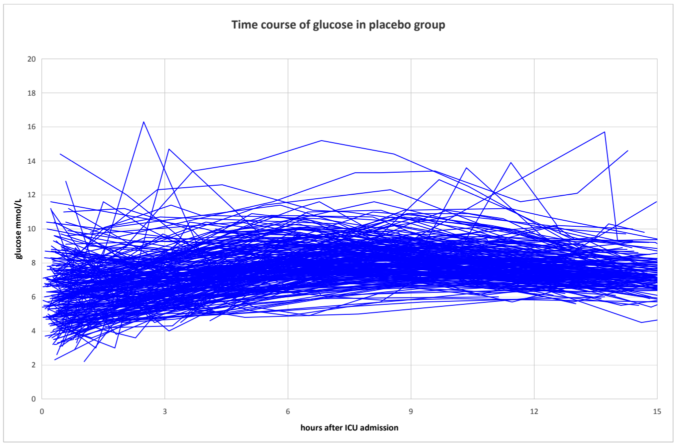
Time course of glucose in placebo group
glucose mmol/L
0 2 4 6 8 10 12 14 16 18 20
0 3 6 9 12 15
hours after ICU admission

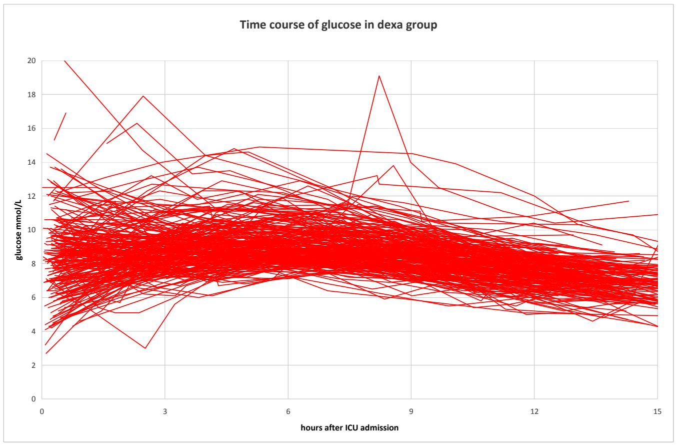
Time course of glucose in dexa group
glucose mmol/L
0 2 4 6 8 10 12 14 16 18 20
0 3 6 9 12 15
hours after ICU admission

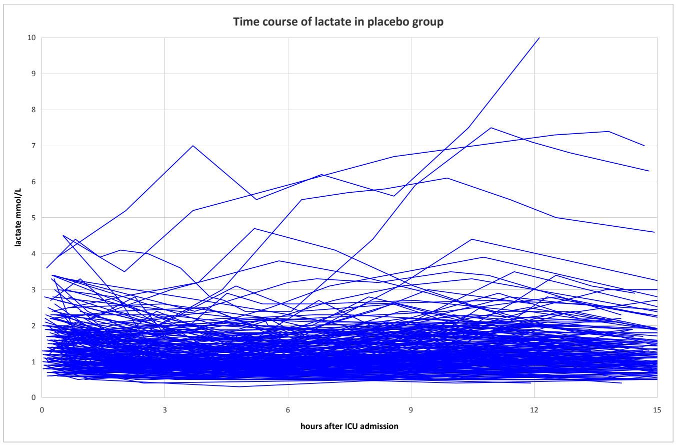
Time course of lactate in placebo group
lactate mmol/L
0 1 2 3 4 5 6 7 8 9 10
0 3 6 9 12 15
hours after ICU admission

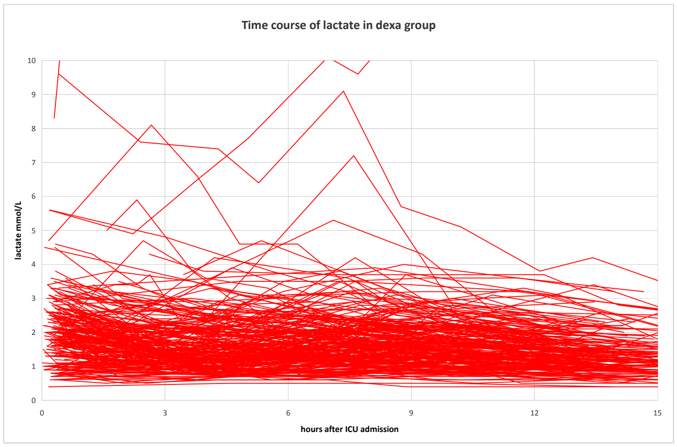
Time course of lactate in dexa group
lactate mmol/L
0 1 2 3 4 5 6 7 8 9 10
0 3 6 9 12 15
hours after ICU admission

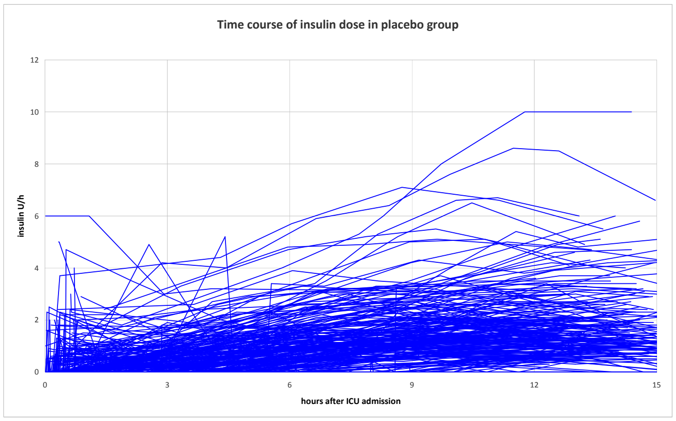
Time course of insulin dose in placebo group
insulin U/h
0 2 4 6 8 10 12
0 3 6 9 12 15
hours after ICU admission

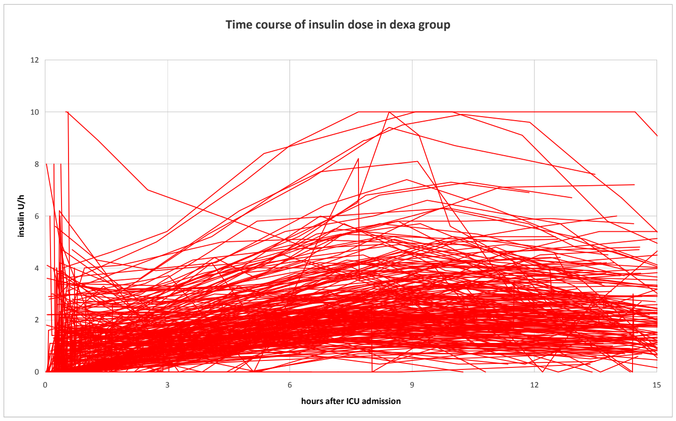
Time course of insulin dose in dexa group
insulin U/h
0 2 4 6 8 10 12
0 3 6 9 12 15
hours after ICU admission

**Additional Table 1: Multivariate linear regression model of the effect of dexamethasone on postoperative lactate levels**				
	**Model 1** R^2**=0.055**		**Model 2** R^2**=0.216**	
	**Beta**	**P-value**	**Beta**	**P-value**
Treatment allocation (to dexamethasone or placebo)	52.711	<.001	12.454	0.208
Glucose area under the curve in the first 15 hours of postoperative ICU stay (AUC_{15}), in mg/dL x h			0.187	<.001
Model dependent variable is lactate area under the curve in the first 15 hours of postoperative ICU stay (AUC_{15}), in mg/dL x h				

Additional Table 2. Bivariate cross-correlation matrix of glucose and lactate levels in the first 15 hours of postoperative ICU stay.

		Glucose															
**Lactate**		**0**	**1**	**2**	**3**	**4**	**5**	**6**	**7**	**8**	**9**	**10**	**11**	**12**	**13**	**14**	**15**
**0**	R	0,238	0,224	0,195	0,222	0,237	0,233	0,212	0,204	0,197	0,134	0,109	,075	,063	,058	,045	,035
	P	,000	,000	,000	,000	,000	,000	,000	,000	,000	,004	,018	,101	,170	,211	,347	,477
**1**	R	0,335	0,355	0,299	0,305	0,305	0,303	0,291	0,282	0,269	0,194	0,153	0,107	,081	,064	,051	,029
	P	,000	,000	,000	,000	,000	,000	,000	,000	,000	,000	,001	,020	,080	,167	,287	,551
**2**	R	0,338	0,361	0,373	0,364	0,343	0,333	0,318	0,308	0,296	0,22	0,166	0,114	,081	,056	,039	,015
	P	,000	,000	,000	,000	,000	,000	,000	,000	,000	,000	,000	,013	,078	,228	,415	,759
**3**	R	0,358	0,403	0,423	0,405	0,368	0,35	0,335	0,328	0,316	0,245	0,179	0,115	,079	,041	,023	-,005
	P	,000	,000	,000	,000	,000	,000	,000	,000	,000	,000	,000	,012	,088	,376	,633	,924
**4**	R	0,367	0,426	0,452	0,432	0,391	0,364	0,345	0,338	0,323	0,256	0,187	0,119	,078	,031	,012	-,019
	P	,000	,000	,000	,000	,000	,000	,000	,000	,000	,000	,000	,009	,088	,501	,801	,691
**5**	R	0,354	0,432	0,467	0,454	0,422	0,385	0,357	0,342	0,321	0,259	0,196	0,129	,085	,032	,010	-,028
	P	,000	,000	,000	,000	,000	,000	,000	,000	,000	,000	,000	,005	,062	,488	,836	,564
**6**	R	0,314	0,403	0,451	0,463	0,453	0,422	0,389	0,367	0,331	0,266	0,203	0,134	0,09	,035	,008	-,037
	P	,000	,000	,000	,000	,000	,000	,000	,000	,000	,000	,000	,003	,049	,451	,858	,443
**7**	R	0,266	0,356	0,405	0,439	0,454	0,438	0,415	0,394	0,349	0,285	0,228	0,162	0,119	,061	,032	-,022
	P	,000	,000	,000	,000	,000	,000	,000	,000	,000	,000	,000	,000	,009	,183	,492	,642
**8**	R	0,224	0,312	0,361	0,41	0,444	0,446	0,439	0,425	0,377	0,32	0,269	0,209	0,166	0,104	,074	,013
	P	,000	,000	,000	,000	,000	,000	,000	,000	,000	,000	,000	,000	,000	,023	,113	,783
**9**	R	0,18	0,269	0,313	0,371	0,418	0,436	0,448	0,449	0,411	0,366	0,321	0,267	0,225	0,159	0,124	,056
	P	,000	,000	,000	,000	,000	,000	,000	,000	,000	,000	,000	,000	,000	,001	,008	,245
**10**	R	0,144	0,222	0,256	0,316	0,368	0,396	0,425	0,443	0,424	0,396	0,364	0,317	0,276	0,209	0,169	0,098
	P	,002	,000	,000	,000	,000	,000	,000	,000	,000	,000	,000	,000	,000	,000	,000	,043
**11**	R	0,113	0,183	0,21	0,27	0,321	0,351	0,388	0,418	0,415	0,409	0,395	0,358	0,32	0,25	0,203	0,127
	P	,014	,000	,000	,000	,000	,000	,000	,000	,000	,000	,000	,000	,000	,000	,000	,009
**12**	R	0,095	0,158	0,185	0,247	0,297	0,323	0,361	0,395	0,397	0,407	0,406	0,384	0,35	0,277	0,226	0,146
	P	,040	,001	,000	,000	,000	,000	,000	,000	,000	,000	,000	,000	,000	,000	,000	,002
**13**	R	,077	0,137	0,165	0,228	0,279	0,303	0,338	0,372	0,374	0,389	0,393	0,38	0,353	0,284	0,235	0,157
	P	,099	,003	,000	,000	,000	,000	,000	,000	,000	,000	,000	,000	,000	,000	,000	,001
**14**	R	,068	0,126	0,158	0,221	0,274	0,294	0,326	0,358	0,356	0,369	0,373	0,365	0,345	0,284	0,24	0,163
	P	,153	,007	,001	,000	,000	,000	,000	,000	,000	,000	,000	,000	,000	,000	,000	,001

eTable 2 (continued). Bivariate cross-correlation matrix of glucose and lactate levels in the first 15 hours of postoperative ICU stay.

**15**	R	,063	0,121	0,153	0,216	0,267	0,289	0,321	0,352	0,348	0,359	0,36	0,353	0,336	0,28	0,242	0,169
	P	,199	,013	,002	,000	,000	,000	,000	,000	,000	,000	,000	,000	,000	,000	,000	,000

Abbreviations: R denotes correlation coefficient, P: P-value.
